# Study protocol for a randomized controlled trial: prophylactic swallowing exercises in head-and-neck cancer patients treated with (chemo)radiotherapy (PRESTO trial)

**DOI:** 10.1186/s13063-020-4171-0

**Published:** 2020-03-02

**Authors:** Margot Baudelet, Leen Van den Steen, Fréderic Duprez, Marc De Bodt, Sarah Deschuymer, Ann Goeleven, Isabel Hutsebaut, Steven Mariën, Sabine Meersschout, Daan Nevens, Sandra Nuyts, Marc Peeters, Pol Specenier, Michiel Van den Brekel, Lisette van der Molen, Caroline Vandenbruaene, Olivier Vanderveken, Joost Van Dinther, Carl Van Laer, Tom Vauterin, Hilde Verstraete, Gwen Van Nuffelen, Member of the Belgian PRESTO Group

**Affiliations:** 1 Department of Radiation Oncology, Ghent University Hospital, Corneel Heymanslaan 10, 9000 Ghent, Belgium; 2 Faculty of Medicine and Health Sciences, Department of Human Structure and Repair, Ghent University, St. Pietersnieuwstraat 33, 9000 Ghent, Belgium; 3 Faculty of Medicine and Health Sciences, University of Antwerp, Universiteitsplein 1 Wilrijk, 2610 Antwerp, Belgium; 4 Department of Otolaryngology and Head and Neck Surgery – Rehabilitation Center for Communication Disorders, Antwerp University Hospital, Antwerp, Belgium; 5 Faculty of Medicine and Health Sciences, Department of Rehabilitation Sciences, Ghent University, St. Pietersnieuwstraat 33, 9000 Ghent, Belgium; 6 Department of Radiation Oncology, KU Leuven, University Hospitals Leuven, Leuven, Belgium; 7 Department of ENT, University Hospitals Leuven, Swallowing Clinic, Leuven, Belgium; 8 Department of Head and Neck Surgery, University Hospitals Leuven, Swallowing Clinic, Leuven, Belgium; 9 Department of Radiation Oncology, AZ Sint-Jan General Hospital, Bruges, Belgium; 10 Department of Radiation Oncology, Iridium Kanker Netwerk, Antwerp, Belgium; 11 Multi-disciplinary Oncological Center Antwerp, Antwerp, Belgium; 12 Laboratory of Experimental Radiotherapy, Department of Oncology, KU Leuven, Leuven, Belgium; 13 Department Medical Oncology, Antwerp University Hospital, Antwerp, Belgium; 14 Department of Head and Neck Oncology and Surgery, Antoni van Leeuwenhoek, Netherlands Cancer Institute, Amsterdam, The Netherlands; 15 Faculty of Humanities, University of Amsterdam, Spui 21, 1012 WX Amsterdam, The Netherlands; 16 Department of Speech-Language Pathology and Audiology, Sint-Jan General Hospital, Bruges, Belgium; 17 Department of Otorhinolaryngology, Sint-Augustinus Hospital, Antwerp, Belgium; 18 Department of Otorhinolaryngology-Head and Neck Surgery, AZ Sint-Jan General Hospital, Bruges, Belgium

**Keywords:** Dysphagia, Head-and-neck cancer, Adherence, Prophylactic swallowing exercises, (Chemo)radiotherapy

## Abstract

**Background:**

Dysphagia is a common and serious complication after (chemo)radiotherapy (CRT) for head-and-neck cancer (HNC) patients. Prophylactic swallowing exercises (PSE) can have a significantly positive effect on post-treatment swallowing function. However, low adherence rates are a key issue in undermining this positive effect. This current randomized trial will investigate the effect of adherence-improving measures on patients’ swallowing function, adherence and quality of life (QOL).

**Methods:**

This ongoing trial will explore the difference in adherence and swallowing-related outcome variables during and after PSE in HNC patients performing the same therapy schedule, receiving different delivery methods. One hundred and fifty patients treated in various hospitals will be divided into three groups. Group 1 performs PSE at home, group 2 practices at home with continuous counseling through an app and group 3 receives face-to-face therapy by a speech and language pathologist. The exercises consist of tongue-strengthening exercises and chin-tuck against resistance with effortful swallow. The Iowa Oral Performance Instrument and the Swallowing Exercise Aid are used for practicing. Patients are evaluated before, during and after treatment by means of strength measurements, swallowing and QOL questionnaires.

**Discussion:**

Since low adherence rates undermine the positive impact of PSE on post-treatment swallowing function, there is need to develop an efficient PSE protocol maximizing adherence rates.

**Trial registration:**

ISRCTN, ID: ISRCTN98243550. Registered retrospectively on 21 December 2018.

## Background

Dysphagia is a common and widely reported complication after (chemo)radiotherapy (CRT) for head-and-neck cancer (HNC) patients and can persist for a long period of time [1–5]. Fifty to 60% of the HNC patients undergoing CRT may experience significant post-treatment dysphagia involving both muscle weakness and incoordination/timing issues [6–8]. The medical consequences (e.g., feeding-tube dependency, malnutrition, aspiration pneumonia) have a major negative impact on daily functioning and health-related quality of life (QOL) and can even be life-threatening [9–15]. These consequences and the high prevalence of swallowing disorders in HNC patients stress the importance of prevention, monitoring and management of this problem [16].

Based on literature and clinical experiences, it can be concluded that nowadays there is no “gold-standard” in the assessment or treatment of dysphagia in HNC patients [17, 18]. An increasing number of studies show that prophylactic swallowing exercises (PSE) can have a significantly positive effect on post-treatment swallowing function, can lead to significantly less muscle atrophy and can improve dysphagia-related QOL in HNC patients treated with CRT [7, 19–24]. However, low adherence rates are a key issue in undermining these positive effects. Reported adherence rates, with adherence defined as “the extent to which patient behavior corresponds with recommendations from a health care provider,” range from 13 to 64% [21, 25–31]. A possible reason for low adherence rates is the additional demand that PSE programs put on the patient, during an already burdensome period [20]. Furthermore, Shinn et al. [25] and Wells and King [30] showed that the etiology can be multifactorial; e.g., forgetting, absence of supervision or the fact that patients do not experience the problem at the start of the exercises.

The indications that PSE can improve patients’ swallowing function and swallowing-related QOL show the crucial, urgent and internationally recognized need for an effective PSE program augmented with measures that may add to adherence [7, 19–25, 27]. Apart from patient characteristics and disease-related aspects, several studies show the impact of different measures on adherence: therapist-supervised exercises, regular counseling and reinforcement sessions, clear and repeated instructions, feedback on successful performance, target setting (e.g., number of repetitions/days) and limited duration [7, 27, 30, 32, 33]. Also, therapist supervision and a close relationship with a therapist play a crucial role in patients’ satisfaction, compliance and individual beliefs in personal skills [30].

Up to now, studies comparing standard PSE with a PSE program augmented with adherence-improving measures are lacking. The randomized controlled trial (RCT) of Wall et al. [26] included 79 patients and investigated whether therapy adherence to prophylactic swallowing exercises was influenced by the delivery method of the exercises. Three different methods were compared. The first group received face-to-face therapy by a speech and language pathologist (SLP), the second group used a telepractice application and the third group practiced independently at home. Adherence was calculated based on the completion of all prescribed exercises. Significantly better adherence rates were found in the group receiving face-to-face therapy and a trend towards better results was found in the group using the application.

This current multicenter randomized trial will investigate the effect of adherence-improving measures on actual patient compliance, swallowing function and QOL. Patient adherence to a prophylactic swallowing-therapy protocol will be examined across three models, similar to the delivery methods in the RCT of Wall et al.: (1) self-help standard PSE (control group), (2) self-help app-supported PSE (app group) and (3) a SLP-supported PSE (therapist group). Our study differs from the above-mentioned study in the type of exercises given, the number of patients included and the adherence-specific measures. The goal of the proposed randomized trial is to develop an optimized PSE program, incorporating patient tolerance and support for the exercise program. The findings of this project should be helpful in setting up future guidelines and directions. The final patient benefit will be improved swallowing function and QOL.

This study aims to

- Conduct a prospective randomized trial investigating the effect of specific adherence measures on patients’ actual compliance, wellbeing, muscle strength, swallowing function and QOL during and following CRT
- Increase insight in the underlying reasons for (non- )adherence in this patient population

## Methods

### Study population

Patients with a stage III or IVA-B (TNM7) newly diagnosed squamous cell carcinoma of the oropharynx are considered as possible participants. Inclusion criteria are: patients treated with radiotherapy or concomitant CRT (CCRT) with or without induction chemotherapy and demonstrating sufficient cognitive and language abilities. The presence of a recurrent carcinoma or metastasis from another carcinoma and previous CRT or surgery in the head-neck region, with possible impact on swallowing function, are exclusion criteria. The patients will be recruited by a radiation oncologist and SLP. The SLP explains the study protocol and study design and assigns participants to the interventions.

### Minimization

All subjects who give consent for participation and who fulfill the inclusion criteria are randomly assigned to one of the three groups with a 1:1:1 allocation by means of the program QMinim. It is an online minimization service supported by the Information and Communication Technology (ICT) Unit of the Antwerp University Hospital. Minimization factors are age (20–60 years vs. ≥ 60 years), treating center, presence of baseline dysphagia and treatment (radiotherapy vs. CCRT).

As this is an open-label trial, the minimization procedure and outcome assessment will not be blinded.

### Study design

The study will be a multicenter RCT. All patients will be training five times a week during the first 4 weeks of CRT. Baseline measurements will be done before the start of CRT. Every week during the training, immediately after CRT and at 1 and 3 months following treatment, patients will be evaluated by radiation therapists and SLPs. Table 1 shows an overview of the study visits and evaluations during the study. Prophylactic swallowing exercises are the same in all groups and comprise evidence-based exercises targeting the main muscle groups involved in swallowing; namely, muscles involved in tongue strength, pharyngeal contraction and laryngeal elevation/upper esophageal sphincter opening. These strengthening exercises include tongue-strengthening exercises (TSE) and chin-tuck against resistance (CTAR) combined with an effortful swallow. First, TSE will be performed since tongue strength is the main bolus-driving force and reduced tongue strength can cause oral and pharyngeal residue and aspiration [34–36]. Second, CTAR exercises are used since they have a significant impact on the suprahyoid muscles, with a positive effect on laryngeal elevation and upper esophageal sphincter opening [37, 38]. The third exercise consists of effortful swallows in combination with the chin-tuck. Effortful swallows have been shown to improve the tongue-base posterior motion and can increase tongue-base pharyngeal-wall pressures [39]. It is hypothesized that the chin-tuck in combination with an effortful swallow stimulates the pharyngeal musculature [38].

**Table 1:** Study visits and evaluations

			Study period				
	Enrollment	Allocation	Post allocation				
Time point	*Pre RT*	*Between enrollment and start RT*	*Weeks 1–4 of RT*	*Weeks 5–7 of RT*	*End of RT*	*1 month after RT*	*3 months after RT*
Enrollment:							
Eligibility screen	X						
Informed consent	X						
Allocation		X					
Interventions:							
*PSE – control group*			X				
*PSE – app group*			X				
*PSE – therapist group*			X				
Assessments:							
*Patient, disease and therapy characteristics*	X						
*Swallowing function*	X		X		X	X	X
*Muscle strength*	X		X		X	X	X
*Impact of mucositis*	X		X		X	X	X
*Quality of life*	X				X	X	X
*Attitudes towards exercises*			X				
*Overall fatigue*	X		X				

*RT* radiotherapy, *PSE* prophylactic swallowing exercises

The different exercises described above alternate during the sessions: all subjects start their first session with TSE and perform CTAR exercises and effortful swallowing in the next session. Tongue-strengthening exercises consist of 120 tongue presses and are divided into 12 sets of 10 repetitions with a 30-s rest between sets and with the target level set at 80% of 1 repetition maximum (1RM, i.e., the maximum amount of pressure that can be generated in one repetition) [34, 40–44]. The next session CTAR exercises and effortful swallows are performed. Each CTAR session consists of 150 chin-tucks against resistance at a target level of 60–70% [45] of the 1RM (i.e., in this study, the maximum chin-tuck strength that can be generated). These chin-tucks are divided into 30 sets of five repetitions with an effortful swallow at every fifth repetition. In both tongue and chin-tuck exercises, a successful repetition was defined as reaching the target level and holding the contraction for 3 s, using the green light (TSE) as biofeedback or tactile biofeedback (CTAR). According to the principle of progressive overload, maximal tongue strength and strength of the suprahyoid muscles and correspondent levels of resistance are measured at baseline and recalculated subsequently every week [44].

All subjects will be randomly assigned into the previously defined groups: control group, app group and therapist group. The groups differ in degree and kind of adherence-improving measures. All groups have some of these measures in common. Restricting the duration to the first 4 weeks of CRT is a first method to increase compliance since the literature shows that in the last 2–3 weeks of therapy the feasibility of completing exercises decreases [20, 33]. Receiving visual and tactile feedback on their performance via the therapeutic devices is a second method. The differences in adherence-improving measures between the groups are illustrated in Table 2. The first group (control group) will perform the exercises at home, without supervision of a SLP but with a counseling session of 10 min every week. Group 2 (app group) practices at home but receives continuous counseling and gets instructions by videos via an application on a tablet, developed in collaboration with Cyborn, Antwerp, Belgium (www.cyborn.be). Generally, mobile health applications are considered to be possible tools to improve traditional health care [46]. By means of gamification, the app helps, supports and motivates the patients to practice. The app registers when the patients practice, how many repetitions they do and when they succeed in doing the exercises. Group 3 (therapist group) is given face-to-face therapy and will be counseled by a SLP five times per week. All patients will complete the first session with supervision of the SLP, irrespective of their group. The intervention will be discontinued on participant request or in case of worsening disease, requiring major changes in treatment. Reasons for discontinuing will be stored in a database.

**Table 2:** Study design

Inclusion	*n* = 150		
Stratified randomization	*n* = 50 Control group	*n* = 50 App group	*n* = 50 Therapist group
Therapy schedule	• 5x/week (30–40 min)	• 5x/week (30–40 min)	• 5x/week (30–40 min)
	• 4 weeks	• 4 weeks	• 4 weeks
	• Home practice	• Home practice but app-supported	• Therapist supervised
Exercises	• TSE	• TSE	• TSE
	• CTAR	• CTAR	• CTAR
	• Effortful swallow	• Effortful swallow	• Effortful swallow
Adherence measurements			
• Supervision	No (home practice)	No (home practice – app)	Yes (face-to-face therapy)
• Counseling	Counseling 1x/week by SLP (10′)	Counseling 1x/week by SLP (10′) & continuous counseling via app	Counseling by SLP 5x/week
• Feedback on performance	Yes – instrumental	Yes – instrumental	Yes – instrumental & by SLP
• Clear and repeated instructions	• Introduction session • Written instructions	• Introduction session • Instructions via app: animation videos	• Each session by the SLP
• Target setting	Yes	Yes	Yes
• Limited duration	Yes – first 4 weeks of CRT	Yes – first 4 weeks of CRT	Yes – first 4 weeks of CRT

*TSE* tongue-strengthening exercises, *CTAR* chin-tuck against resistance, *SLP* speech and language pathologist, *CRT* (chemo)radiotherapy

### Instrumentation

**Fig. 1:**
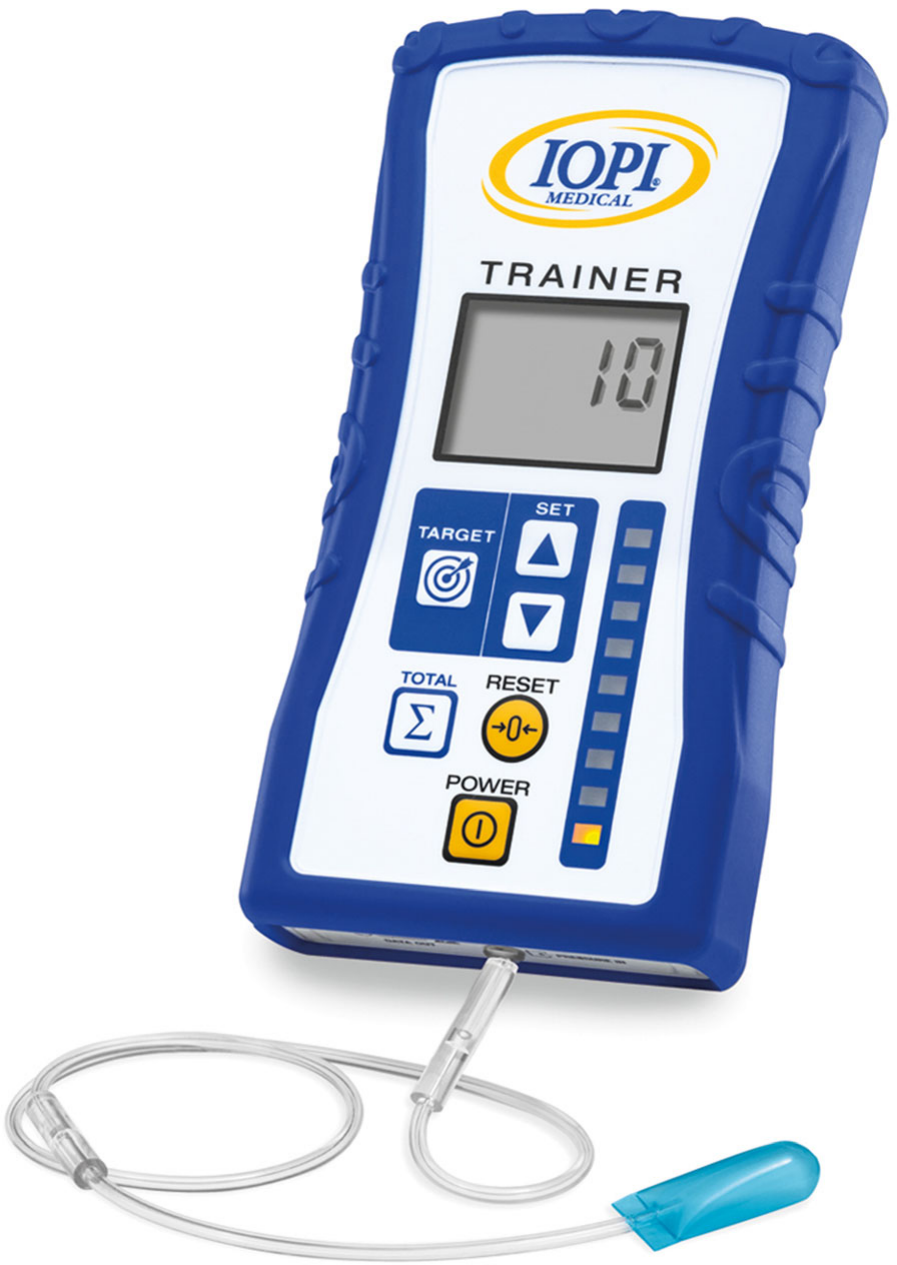
Iowa Oral Performance Instrument, model 3.2 (IOPI Medical LLC, Woodinville, WA, USA)

*Maximal tongue strength* is measured by the Iowa Oral Performance Instrument (IOPI) Pro, model 3.1 (IOPI Medical LLC, Woodinville, WA, USA). This is a small, portable instrument connected to an air-filled bulb. Maximal isometric pressure can be measured anteriorly and posteriorly, MIP_a_ and MIP_p_, respectively. The digital display shows the amount of pressure that the tongue produces (in kilopascal, kPa) when squeezing the bulb against the palate. In the anterior position, the proximal end of the bulb is placed immediately behind the upper teeth at the midline of the palate. In the posterior position, the bulb is placed at the level of the transition from the hard to the soft palate. The subjects are instructed to push the bulb as hard as possible against the palate. The highest value of three trials is considered the MIP. Tongue-strengthening exercises are done using the IOPI Trainer, model 3.2 (Fig. 1), which is similar to model 3.1. The device allows the therapist to set a target manually. There is a vertical series of small lights providing visual feedback: the upper light becomes green when the subject reaches the target. The TSE only consists of anterior tongue presses since previous research shows that the increase in tongue strength depends on the localization of the bulb: higher increases of anterior and posterior tongue strength are obtained when training exclusively anteriorly [47]. Patients are instructed to squeeze the bulb against the hard palate until the upper light becomes green and are asked to hold this effort for 3 s [48].

**Fig. 2:**
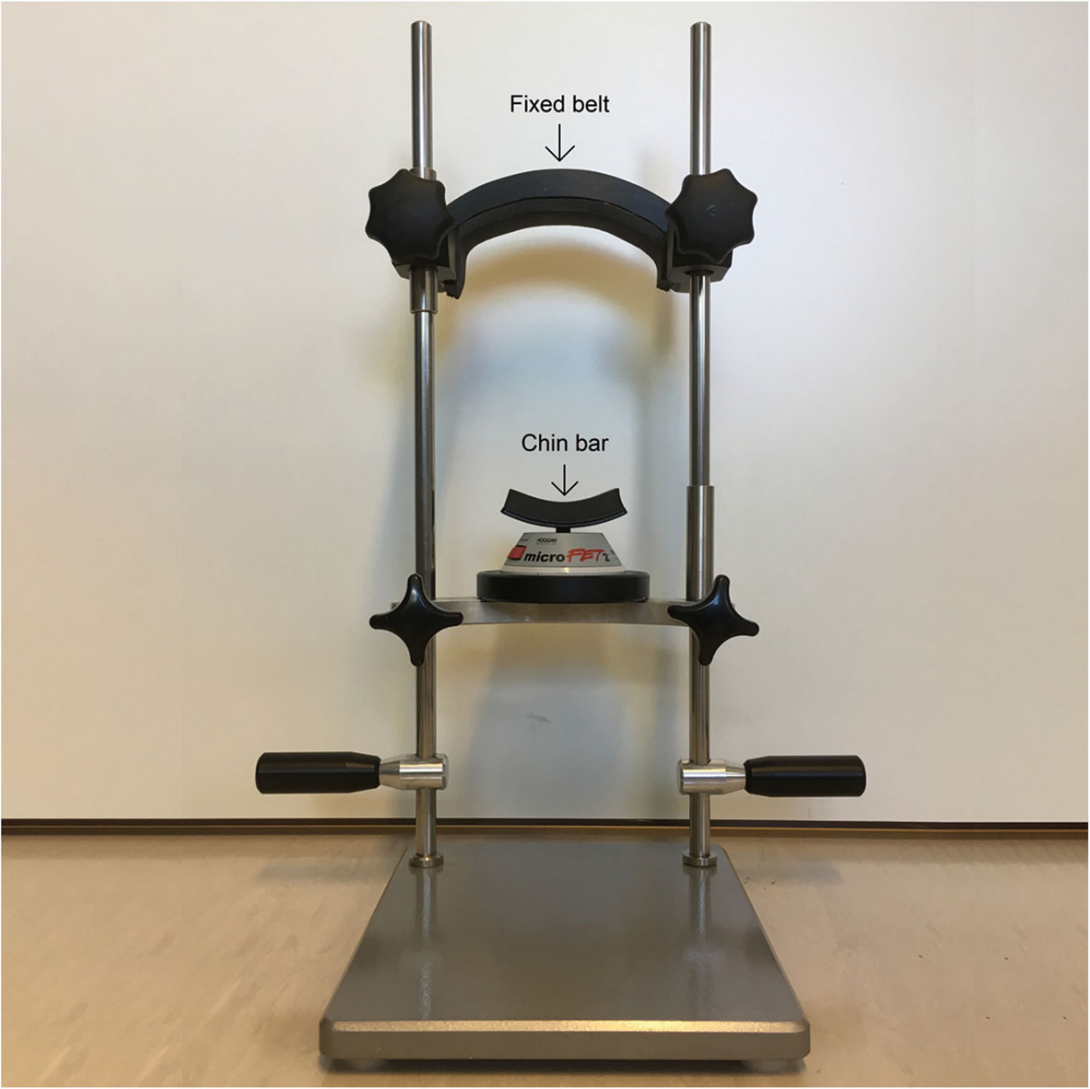
Examination frame and dynamometer (MicrofetTM, Biometrics, Almere, The Netherlands)

**Fig. 3:**
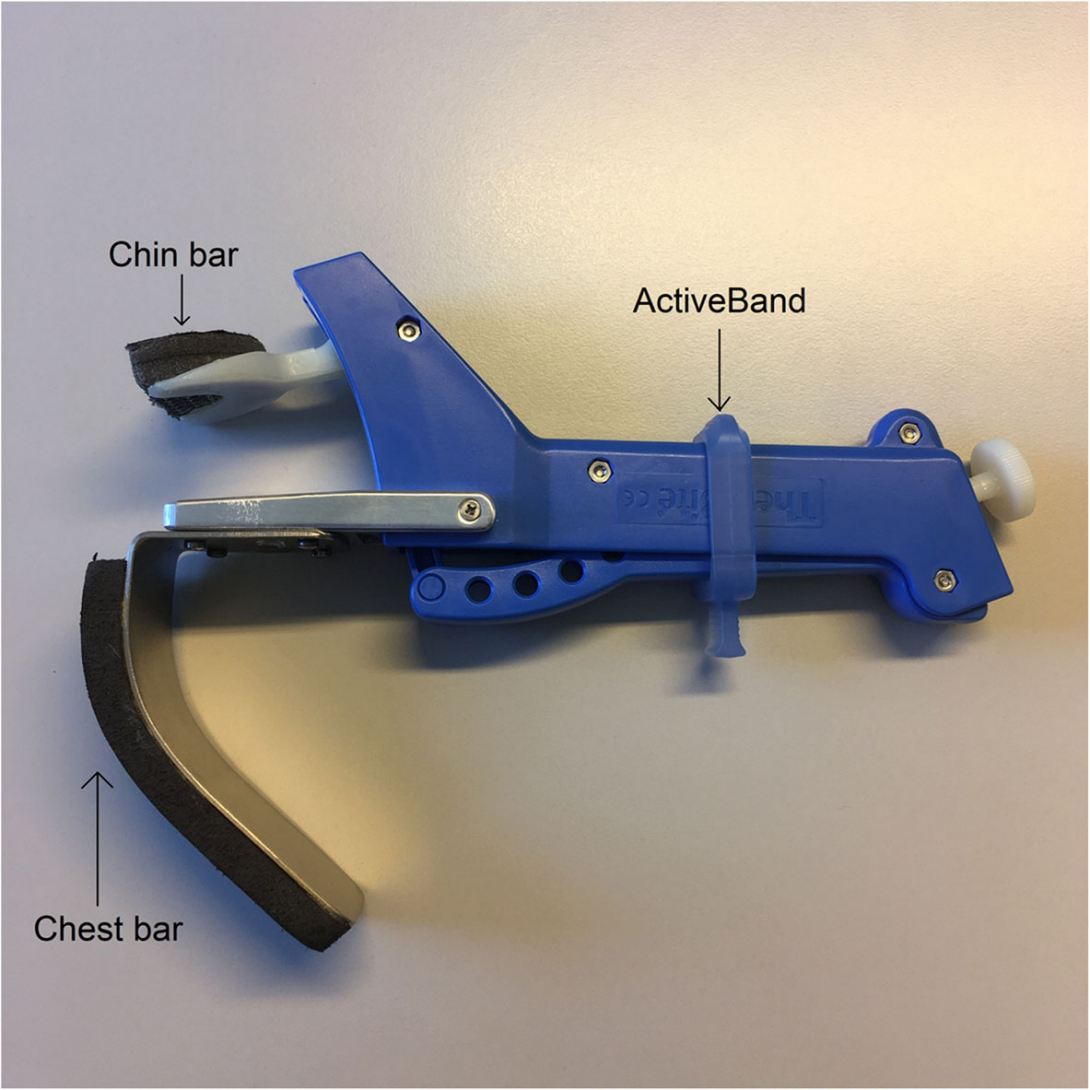
Swallowing Exercise Aid (SEA)

*Maximal chin-tuck strength* (in Newtons, N) is measured by means of a dynamometer (Microfet™, Biometrics, Almere, The Netherlands) (Fig. 2). Patients are asked to place their chin on the chin bar and keep their mouth and teeth closed. A fixed belt stabilizes the patients’ head. They are instructed to press their chin down as hard as possible. The highest value of three trials is considered the maximal isometric chin-tuck strength. Based on this value, the resistance level can be assessed using the conversion table from Kraaijenga et al. (value from 1 to 6) [38, 45]. The CTAR exercises are performed using the Swallowing Exercise Aid (SEA) (Fig. 3). It is a medical device remodeled by a technician of the Netherlands Cancer Institute by adding a chest bar to one of the mouthpieces of the commercially available TheraBite Jaw Mobilization device (Atos Medical, Hörby, Sweden) [38]. The ActiveBand can be placed at various, marked positions depending on the desired resistance. The minimal resistive load is 4 N (position 1) and the maximal resistive load is 50 N (position 6). Subjects are asked to hold the device with one hand, place the chest bar against the chest and put their chin on the chin bar. They are instructed to press the chin bar towards the chest bar and hold this effort for 3 s. At every fifth repetition, patients are asked to press the chin bar against the chest bar and swallow as hard as they can (effortful swallow).

### Outcome measures

The primary outcome measure of this study is the swallowing function, based on scores of the Mann Assessment of Swallowing Ability-Cancer (MASA-C) [49]. Next to this measurement, patients are asked to fill out the Eating Assessment Tool-10 [50] and a Visual Analog Scale. The Functional Oral Intake Scale [51] is filled in by a SLP. The swallowing function is assessed at baseline, weekly during the weeks of exercise, immediately after CRT and 1 and 3 months after CRT (Table 1).

There are three secondary outcome measures: (1) degree of compliance, (2) muscle strength and (3) QOL.

The degree of compliance is expressed as the total number of exercises performed per week, based on daily patient (group 1 and 2) and therapist (group 3) registration. The number of performed tongue-strengthening exercises is recorded automatically by means of the IOPI. In group 2, the degree of compliance is also expressed by the time spent on the app, which is registered automatically. The compliance is assessed during the first 4 weeks of CRT. MIP_a_, MIP_p_ and strength of the suprahyoid muscles are examined at the same time as the swallowing function (Table 1) and are measured using the IOPI and a dynamometer. The Swallowing Quality-of-Life Questionnaire [52] and the Dysphagia Handicap Index [53] examine the swallowing-related QOL and are assessed at baseline, at the end of CRT and 1 and 3 months after CRT (Table 1).

All these evaluations are performed by a SLP.

### Confounders

#### Patient, disease and therapy characteristics

Patient and situational characteristics are questioned at baseline and include age, gender, educational level, social status, experience with mobile phones, tablets, etc. and the presence of support from family or friends. The question about the experience with mobile phones and tablets is an open question that allows the patients to answer extensively. The question about the support is a closed question: yes/no and who. The NEO Five Factor Inventory is used to examine the personality of the patient [54]. The disease and therapy characteristics include location of the carcinoma, stage, treatment, fractionation, TNM classification (TNM7) and human papillomavirus (HPV) status. This information is gained by radiation oncologists, head and neck surgeons and otolaryngologists.

#### Evaluation of the impact of mucositis

The Oral Mucositis Weekly Questionnaire [55] is used to measure the symptoms of mucositis and the impact on the patient’s well-being and function.

#### Evaluation of attitudes towards exercising, motivation and fatigue

The attitudes on exercises are surveyed by the questionnaire of Sluijs, Kok and Van der Zee [56] and the overall fatigue of the patients is evaluated by the Multidimensional Fatigue Inventory [57] (Table 1).

### Data management and monitoring

The datasets generated during the study will be stored in a non-publicly available repository. All clinical record forms are collected and managed using REDCap (Research Electronic Data Capture) electronic data capture tools hosted at Ghent University Hospital [58]. This is a secure, web-based application designed to support data capture for research studies. All patient information (except identifying information), questionnaires and measurements are stored for 20 years. The researchers from each participating institution have access to the data of their patients. All data is pseudonymized and patients’ details are encoded. The principle investigator and the researcher at the University Hospital in Ghent manage the entire database.

In order to avoid introducing bias, no interim analyses will be performed. As the experimental interventions caries minimal risks, no data monitoring committee will be implemented, nor will there be a stopping procedure.

There is no anticipated harm and so no compensation for trial participation. After completing the trial, participants will be followed by the radiation oncologist and head and neck surgeon or otolaryngologist and, if necessary, they will be referred to any other specialist.

Serious adverse events will be reported to the Ethical Committee of the central study center by means of a yearly line-listing system.

### Statistical analysis

#### Sample size calculation

The sample size calculation is performed using GLIMMPSE32 and based on published data on MASA-C scores [49]. A total sample size of 111 participants (37 per group), is needed to demonstrate a different evolution over time in the experimental groups and the control group at a significance level of 0.05 and a power of 0.8 when using repeated measures with Geisser-Greenhouse correction (3 groups × 4 time points). Based on previous research on prophylactic swallowing exercises by Carnaby-Mann and colleagues [19], we expect a baseline MASA-C score of 195, with a decay in the first 6 weeks to 171 in the control group, 177 in the app group and 180 in the therapist group. After 6 weeks we assume no further decline. A standard deviation of 11 is considered for the MASA-C scores at each time point, this is also based on the standard deviations (SDs) reported in Carnaby-Mann et al. [19]. First-order auto-regressive correlation (AR [1]) is assumed for the covariance structure between the repeated measures, with a base correlation of 0.4 and a decay rate of 0.5. The targeted total sample size, taking into account more than 20% dropouts, is 150 (*n* = 50/group).

#### Data analysis

Patients will be analyzed in the groups to which they are assigned (intention-to-treat). Descriptive statistics will be used to summarize patient characteristics per treatment group. Data will be analyzed using linear mixed-effects models with group, time and group by time interaction as fixed effects. A random intercept will be added to account for correlation between measurements coming from the same individual. When one or more overall effects are significant, post-hoc pairwise testing with Bonferroni-Holm correction for multiple testing will be performed. Missing data is assumed to be missing at random (MAR) and, thus, will be ignored in the analyses. By using mixed-effects models for the analysis, we can incorporate all information on the available time points. If more than 15% of the data is missing, a sensitivity analysis will be conducted by using multiple imputation. Results of the original analysis of the available cases will be interpreted in the context of sensitivity analysis.

Additionally, the impact of adherence, HPV status, dosimetry and mucositis on the different functional endpoints will be studied in exploratory linear mixed-effects models. Fixed main-effects and interactions with group for all these factors will be evaluated.

More details on the primary, secondary and exploratory analyses can be found in the statistical analysis plan.

A *p* value of less than 0.05 will be considered statistically significant. All analyses will be conducted using SPSS Statistics version 25 (IBM, Chicago, IL, USA) and R software (R Foundation, Auckland, New Zealand).

### Patient and public involvement

Pre-recruitment there has been a try-out with a small number of patients. They were questioned about the feasibility of the exercise protocol. Also, during the study all participants are asked about their experiences regarding the exercises. In the context of the application, all patients are questioned about their familiarity with smartphones, tablets and computers. Before the start of the study, the application has been tested by a number of people from different age groups.

### Study sites

This multicenter study will be conducted at the following sites: Antwerp University Hospital, Ghent University Hospital, University Hospital Leuven, Academic Hospital Sint-Jan Bruges, Sint-Augustinus Antwerp and other partners of the Iridium Cancer Network.

All participating centers have extensive experience in research on head-and-neck cancer and dysphagia. Data collection in these hospitals enables the study to include enough patients and gives the study sufficient power.

## Discussion

An increasing number of studies have shown a significantly positive effect of PSE on swallowing function in HNC patients treated with CRT. However, low adherence rates are a major issue, preventing clinical implementation of PSE. There is an internationally recognized need to develop the most efficient PSE protocol in which the adherence rates are maximized. This multicenter randomized trial will investigate the effect of adherence-improving measures on actual patient compliance, swallowing function, muscle strength and QOL. It is expected that this study will result in an optimized, patient-supported and evidence-based PSE program improving patient compliance.

## Data Availability

The datasets generated during the current study are not publicly available since they will contain patient data and the informed consent does not include sharing-data publicly. The datasets are available from the corresponding author on reasonable request. All clinical record forms will be collected and managed using REDCap (Research Electronic Data Capture) electronic data capture tools hosted at Ghent University Hospital [58].

## Abbreviations

1RM: 1 repetition maximum
AR (1): First-order auto-regressive correlation
CCRT: Concomitant chemoradiotherapy
CRT: (Chemo)radiotherapy
CTAR: Chin-tuck against resistance
HNC: Head-and-neck cancer
HPV: Human papillomavirus
IOPI: Iowa Oral Performance Instrument
MAR: Missing at random
MASA-C: Mann Assessment of Swallowing Ability–Cancer
MIP_a_: Anterior maximal isometric pressure
MIP_p_: Posterior maximal isometric pressure
PSE: Prophylactic swallowing exercises
QOL: Quality of life
RCT: Randomized controlled trial
REDCap: Research Electronic Data Capture
SEA: Swallowing exercise aid
SLP: Speech and language pathologist
TSE: Tongue-strengthening exercises
